# Hypoxia in the pulmonary vein increases pulmonary vascular resistance independently of oxygen in the pulmonary artery

**DOI:** 10.1002/ame2.12402

**Published:** 2024-03-20

**Authors:** Sigridur Olga Magnusdottir, Carsten Simonsen, Dan Stieper Karbing, Bodil Steen Rasmussen, Benedict Kjaergaard

**Affiliations:** ^1^ Biomedical Research Laboratory Aalborg University Hospital Aalborg Denmark; ^2^ Department of Clinical Medicine Aalborg University Aalborg Denmark; ^3^ Department of Cardiothoracic Surgery Aalborg University Hospital Aalborg Denmark; ^4^ Department of Health Science and Technology Aalborg University Aalborg Denmark; ^5^ Department of Anesthesia and Intensive Care Aalborg University Hospital Aalborg Denmark

**Keywords:** animal models, cardiopulmonary support, Hypoxic pulmonary vasoconstriction, pulmonary circulation

## Abstract

**Introduction:**

Hypoxic pulmonary vasoconstriction (HPV) can be a challenging clinical problem. It is not fully elucidated where in the circulation the regulation of resistance takes place. It is often referred to as if it is in the arteries, but we hypothesized that it is in the venous side of the pulmonary circulation.

**Methods:**

In an open thorax model, pigs were treated with a veno‐venous extra corporeal membrane oxygenator to either oxygenate or deoxygenate blood passing through the pulmonary vessels. At the same time the lungs were ventilated with extreme variations of inspired air from 5% to 100% oxygen, making it possible to make combinations of high and low oxygen content through the pulmonary circulation. A flow probe was inserted around the main pulmonary artery and catheters in the pulmonary artery and in the left atrium were used for pressure monitoring and blood tests. Under different combinations of oxygenation, pulmonary vascular resistance (PVR) was calculated.

**Results:**

With unchanged level of oxygen in the pulmonary artery and reduced inspired oxygen fraction lowering oxygen tension from 29 to 6.7 kPa in the pulmonary vein, PVR was doubled. With more extreme hypoxia PVR suddenly decreased. Combinations with low oxygenation in the pulmonary artery did not systematic influence PVR if there was enough oxygen in the inspired air and in the pulmonary veins.

**Discussion:**

The impact of hypoxia occurs from the alveolar level and forward with the blood flow. The experiments indicated that the regulation of PVR is mediated from the venous side.

## Graphical abstract

A ventilator and veno‐venous extracorporeal membrane oxygenation (VV‐ECMO) are used for improving oxygenation in the daily clinic, but experimentally they can be used to remove oxygen.

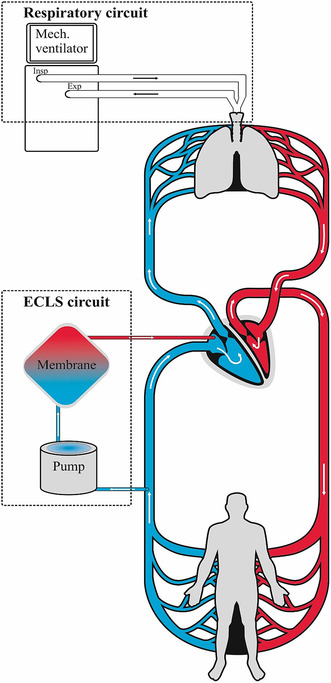

## 1 INTRODUCTION

If parts of the lungs are deprived of oxygen, for example, due to pneumothorax or atelectasis, blood flow will be directed to better‐ventilated areas to protect the body from low arterial oxygen tension as a consequence of shunting blood through poorly ventilated parts of the lungs. This mechanism, called hypoxic pulmonary vasoconstriction (HPV), is one of the most significant physiological mechanisms regulating ventilation/perfusion matching in the lungs. In a way, the same protective mechanism of changes in pulmonary vascular resistance (PVR) can be both beneficial and harmful, and the patient might end up in a vicious, self‐augmenting cycle.^1^

It is still not fully elucidated where the regulation of resistance occurs in the pulmonary circulation. In the systemic circulation, the arterioles are the major site of resistance, and hyperoxia results in vasoconstriction.^2^ In the pulmonary circulation, oxygen has the opposite effect on vascular resistance, and hypoxia leads to HPV and thereby impairs pulmonary gas exchange in the regions where perfusion is abolished.^3^ If pulmonary embolism results in high PVR, it seems clear that the cause is in the arteries, but, for many other causes, including hypoxia, the literature regarding the cause is more unclear. Most articles refer to the pulmonary arterioles as the site of resistance to HPV.^4^, ^5^, ^6^, ^7^ A few articles refer to the pulmonary veins as the site responsible for HPV, either due to constriction in the venules or as the triggering site from which signals are sent to the arteriolar site.^4^, ^5^, ^6^, ^7^, ^8^, ^9^, ^10^

Extracorporeal membrane oxygenation (ECMO) has been used in a variety of clinical and experimental situations, helping humans and animals with severe hypoxia in different setups, for instance, in patients suffering from COVID‐19, pigs with carbon monoxide poisoning, and both animals and humans suffering an otherwise‐fatal pulmonary embolism.^11^, ^12^, ^13^, ^14^, ^15^ The pig is a well‐suited model for humans, because both the anatomy and physiology resemble those of humans, and pigs are known to have a strong pulmonary vasoconstriction response to various stimuli, such as hypoxemia.^16^, ^17^

**FIGURE 1:**
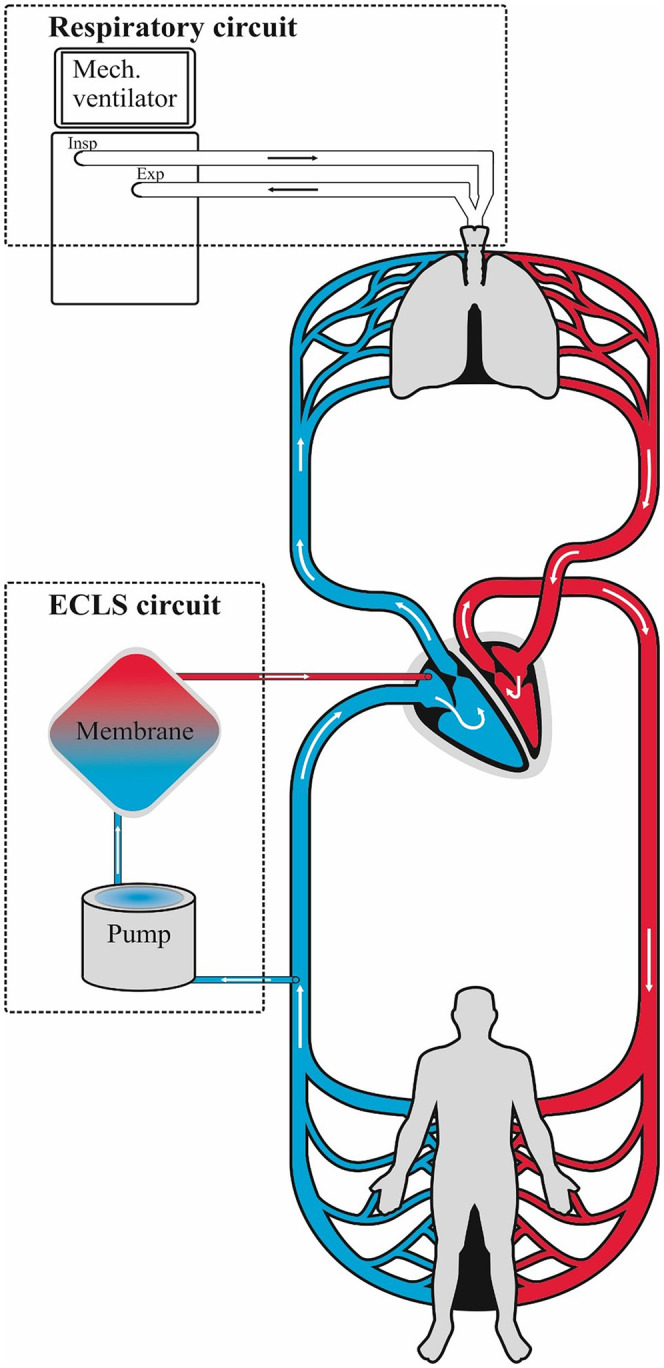
A schematic drawing of a normal veno‐venous extracorporeal membrane oxygenation (ECMO) setup in humans, showing deoxygenated blood going from the patient and into the ECMO oxygenator, which delivers oxygenated blood to the patient at the same time as the ventilator delivers oxygen directly to the lungs.

In the human clinic, treatment with ECMO in severe hypoxia is most often with the use of a veno‐venous (VV) circulation through an oxygenator delivering oxygen (O_2_) and removing carbon dioxide (CO_2_) as a supplement to the ventilator (Figure 1). In this porcine experiment we used the same system, but, in a setup, we also removed O_2_ from the blood with the oxygenator or removed O_2_ from the airway using a ventilator with a very low fraction of inspired air (FiO_2_) and to some extent changing oxygenation in either the pulmonary artery or in the pulmonary veins.

The aim of the study was to elucidate where hypoxia triggers HPV in the pulmonary system, using the setup described earlier, allowing unprecedented control of pulmonary arterial and venous O_2_ and CO_2_.

## 2 MATERIALS AND METHODS

### 2.1 Ethics Statement

The experiments were carried out according to Danish and European legislation. The experiments were approved by the Danish Animal Experiment Inspectorate, license no. 2016‐15‐0201‐00930, and were in line with the Utstein recommendation for uniformity in animal experiments.^18^

### 2.2 Animal care

Twelve female Danish Landrace pigs were used for the experiments. The animals' median weight was 41.5 kg. The age of the animals was 3–4 months. They all came from the same specific pathogen‐free farm. The animals were allowed 1 week of acclimation after arrival in the experimental facilities. They were housed in pairs and were fed standard feed (Altromin, Altromin Spezialfutter, Lage, Germany). The pigs were fasted for 12 h before the experiments but had access to water.

The animals were premedicated with an intramuscular injection of Zoletil Vet (Virbac Danmark, Kolding, Denmark) (1 mL/10 kg), a mixture of two dissociative anesthetics (ketamine, 8.3 mg/mL, and tiletamine, 8.3 mg/mL), a benzodiazepine (zolazepam, 8.3 mg/mL), a synthetic opioid (butorphanol, 1.7 mg/mL), and an alpha 2‐adrenergic agonist (xylazine, 8.3 mg/mL). The animals were then transferred to the operating room where they were intubated through the trachea (Portex tube, ID 6.5 mm, Smiths Medical, UK) and connected to a ventilator (Dameca DREAM, Roedovre, Denmark). The tidal volume was set to 8 mL/kg, and the FiO_2_ was set to 0.60. The respiratory rate (RR) was adjusted to maintain end‐tidal carbon dioxide (EtCO_2_) at 4.5–5.5 kPa. To avoid atelectasis, positive end‐expiratory pressure (PEEP) was set at 5 cm H_2_O, and recruitment was performed regularly, where PEEP was increased to 10–20 cm H_2_O with a breathing frequency of 10 breaths per minute and an inspiration–expiration ratio of 1:1 for 1 min each time.

Anesthesia was maintained with a continuous intravenous infusion of propofol (4 mg/kg/h) and fentanyl (5 μg/kg/h), and the infusion rates were adjusted to maintain sufficient anesthesia. Monitoring with electrocardiogram and peripheral oxygen saturation (SpO_2_) was established. A bladder catheter with a thermal sensor (Foley Catheter, 10Ch/FR, Unomedical, Kedak, Malaysia) was inserted. Arterial pressure measurements were established with a catheter in the left femoral artery (6 Fr, Avanti, Cordis Cashel, Ireland). The same catheter was used for arterial blood gas sampling.

During the entire experiment, no vasoactive drugs were used.

### 2.3 Exclusion criteria

The exclusion criteria were temperature below 36°C, PVR >1000 dyn s/cm^5^, and/or cardiac output (CO) reduced to more than half of baseline CO, because CO output would impact PVR, but the change would not necessarily be due to increased resistance in the pulmonary circulation. Animals with severe cardiac arrhythmias requiring medication were excluded.

### 2.4 Experimental setup

After heparinization (30 000 IU (international units) initial doses), VV circulation was established with a 15 Fr cannula (Medtronic Inc., Minneapolis, MN, USA) in the superior caval vein inserted via the right jugular vein and a 22 Fr cannula (Sorin Group Italia, Mirandola, Italy) in the inferior caval vein inserted via a femoral vein using percutaneous techniques. A prototype centrifugal pump was used to drive the extracorporeal circulation through an oxygenator (QUADROX adult, Maquet, Rastatt, Germany). The VV blood flow in the extracorporeal circuit unit was monitored with an ultrasonic flow meter (Sono TT, em‐tec GmbH, Finning, Germany).

As mentioned in a daily clinical situation, this type of oxygenator is used only for delivering oxygen to the blood and for removing an adequate amount of CO_2_. In our experimental setting, we utilized its ability to also remove oxygen from the blood when the oxygenator sweep air flow had very low oxygen tension.

**FIGURE 2:**
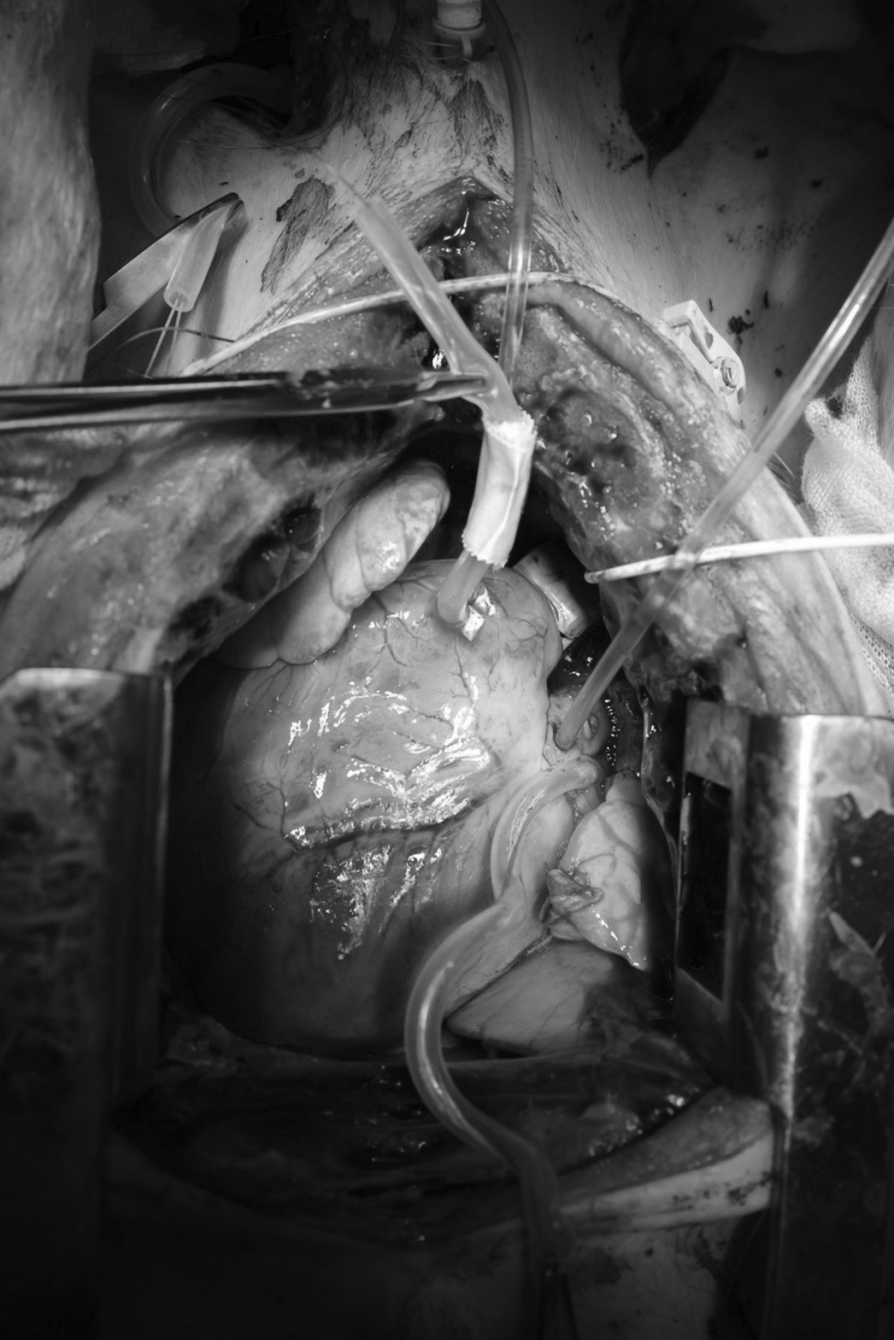
The ultrasonic probe around the pulmonary artery and the pressure probes in the left atrium and pulmonary artery.

A sternotomy was performed. To minimize blood sieving from the incision, bone wax was applied to the sternum. Small catheters were inserted into the pulmonary artery and left atrium to measure pressure differences throughout the pulmonary vascular system (Figure 2).

The catheter in the pulmonary artery was also used for withdrawing mixed venous blood for analysis. Using an articulating dissection instrument (Wolf Lumitip Dissector, AtriCure, Mason, OH, USA) modified for multiple uses as a guide, the fibrous tissue connecting the aorta and the pulmonary artery was divided, allowing the placement of a sonography probe (16 mm, Medistim, Copenhagen, Denmark) around the main pulmonary artery. The probe was connected to a flow monitor (Medistim), enabling continuous measurements of CO, used to calculate PVR.

### 2.5 Experimental protocol

The experiments were performed in two parts, each part using six individual animals and a special setup, affecting oxygenation either in the pulmonary artery or in the pulmonary vein. It was not possible to use the same pigs for both parts. The partial pressure of CO_2_ was held within the normal range as far as possible. Before new interventions, the animals were allowed to reach a steady state with stable values of oxygen and carbon dioxide tensions in the blood for at least 15 min.

#### 2.5.1 Part 1: Changing oxygen tension in the pulmonary veins

After all the previously described catheters and probes were placed, the animal was allowed to reach a steady state, and arterial and mixed venous gas was drawn for baseline values. When the baseline blood samples were taken, the animals were on a ventilator with FiO_2_ 0.60 and with no ECMO intervention.

When the steady state had been confirmed with blood gas samples, FiO_2_ on the ventilator was adjusted in two steps, first from 0.60 to 1.00 and thereafter stepwise down. After every adjustment, the animal was allowed to reach a steady state before new arterial blood gas and mixed venous blood samples were drawn at the same time as all data (blood pressure [BP], mean pulmonary artery pressure [PAP], mean left atrial pressure [LAP], heart rate [HR], RR, FiO_2_, CO, and temperature [*T*]) were registered. For each adjustment, the PVR was calculated from the following equation:

$$\mathrm{PVR}=\left(\mathrm{PAP}-\mathrm{LAP}\right)\times 80\;\mathrm{CO}$$

After increasing to FiO_2_ = 1.00, the same stepwise procedure was repeated for reductions in FiO_2_ down to 0.60, 0.21, 0.15, 0.10, and 0.05. By doing so, it was possible to map the exact changes in PVR during changes in FiO_2_ and thereby of oxygen in the pulmonary veins and thus partial pressure of arterial oxygen (PaO_2_).

To reduce the FiO_2_ to 0.05, a special setup was made for the ventilator, where the air supply to the ventilator was replaced with nitrogen.

Before the FiO_2_ was set below 0.21, VV‐ECMO was started. The pump was set to 2700 rpm, resulting in a mean flow of 2.99 L/min (standard deviation [SD]: 0.339 L/min). After the animal was coupled to the extracorporeal circulation and before the study was continued, the animal was allowed to reach a steady state, which was verified by drawing venous and arterial blood gas samples, used as new baseline values for the rest of the experiment.

Once the animal was supported by VV‐ECMO with the special ventilator setup, the FiO_2_ was decreased as low as 0.05. This made it possible to supply the animal with oxygen to the pulmonary arteries and to remove oxygen from the lungs with the help of the ventilator and so reduce oxygen tension in the pulmonary veins. When the whole spectrum of FiO_2_ was reached, the animal was euthanized with an intravenous overdose of pentobarbiturate (Euthasol, 400 mg/mL, Virbac Danmark A/S, Kolding, Denmark). The whole experiment took about 8 h to perform.

#### 2.5.2 Part 2: Changing oxygen tension in the pulmonary artery

After all the previously described catheters and probes were placed, the animal was allowed to reach a steady state where PaO_2_ was 10.5–13.5 kPa and PaCO_2_ was 4.5–5.8 kPa.

When the steady state, lasting at least 15 min, had been confirmed with arterial and mixed venous blood gas samples, extracorporeal circulation was started. Before the study was continued, the animal was again allowed to reach a steady state, verified by new arterial blood and mixed venous gas samples. The gas supply to the oxygenator in the extracorporeal circulation was then replaced with a mixture of nitrogen and carbon dioxide. The flow was set to 13–14 L nitrogen and 0.75–1.0 L CO_2_, and CO_2_ was adjusted to maintain normal arterial CO_2_ values. The ECMO pump was set to 2800 rpm, resulting in a mean flow of 3.05 L/min (SD: 0.595). New arterial blood gas and mixed venous blood samples were drawn every 15 min, and all data (BP, PAP, LAP, HR, RR, FiO_2_, CO, and *T*) were registered. Every time a blood gas sample was drawn, the PVR was calculated.

This setup was allowed to run for 1 h, allowing the tracking of changes in the PVR during the period. After 1 h, the animal was euthanized with an intravenous overdose of pentobarbiturate (Euthasol, 400 mg/mL, Virbac Danmark A/S).

### 2.6 Statistical analysis

The resource equation was used to determine the sample size as follows: *E* = (the total number of experimental units) − (the number of treatment groups), where *E* should be between 10 and 20 animals.

To minimize the number of animals in our experiment, we chose six animals per group, and thus *E* = 12–2 = 10.^19^

The statistical analysis was performed in IBM SPSS Statistics software (version 26, SPSS Inc., Armonk, NY, USA). The Shapiro–Wilk test was used to test data for normal distribution. Comparisons of groups at baseline were performed with independent sample *t*‐tests. Comparisons of values before and after changes in oxygenation or after the establishment of ECMO were performed with paired sample *t*‐tests. In cases lacking a normal distribution, the Wilcoxon signed‐rank test was used. Two‐sided *p* < 0.05 was considered statistically significant.

## 3 RESULTS

Data were normally distributed, and there were no statistically significant differences between the study groups at baseline (Table 1).

**TABLE 1:** Baseline characteristics after surgery before establishing ECMO.

	Hyper‐ and hypoxia in the pulmonary vein^a^	Hypoxia in the pulmonary artery^a^	Independent *p*‐value‐test
Weight (kg)	46.37 (5.81)	41 (1.37)	0.052
PVR (dyn s/cm^5^)	197.36 (61.32)	221.29 (77.27)	0.566
pH	7.424 (0.065)	7.408 (0.08)	0.704
PCO_2_ (kPa)	5.24 (0.64)	5.27 (0.68)	0.952
PO_2_ (kPa)	30.02 (10.21)	31.77 (7.86)	0.746
PvO_2_ (kPa)	6.57 (1.13)	6.19 (0.4)	0.457
*T* (°C)	37.67 (0.63)	37.05 (0.97)	0.218
CO (L/min)	4.02 (0.66)	3.35 (0.58)	0.089
PAP (mmHg)	20 (2.97)	18 (4.23)	0.263
Hb (mmol/L)	5.13 (1.07)	5.62 (0.45)	0.332
K+ (mmol/L)	3.78 (0.21)	3.85 (0.55)	0.788
Lactate (mmol/L)	0.88 (0.25)	0.88 (0.27)	1000
MAP (mmHg)	107 (24.11)	102 (11.11)	0.613
HR (beats/min)	70 (8.94)	67 (11.52)	0.664
LAP (mmHg)	10 (2.34)	8 (2.32)	0.318

Abbreviations: CO, cardiac output; ECMO, extracorporeal membrane oxygenation; Hb, hemoglobin; HR, heart rate; LAP, mean left atrial pressure; MAP, mean arterial pressure; PAP, mean pulmonary artery pressure; pH, arterial pH; PVR, pulmonary vascular resistance; *T*, temperature.

^a^ Mean (standard deviation [SD]).

Baseline values were measured after the surgical procedures and again after the animal had been coupled to VV‐ECMO. All measured values were within the normal ranges and were not significantly different after VV‐ECMO except for PVR, PaO_2_, and temperature where ECMO alone influenced the measured values (Table 2).

**TABLE 2:** Baseline characteristics after establishing ECMO.

	Before ECMO^a^	After ECMO^a^	*p*‐value
PVR (dyn·s/cm^5^)	209.33 (67.67)	290.54 (100.12)	0.03
PaO_2_ (kPa)	30.89 (8.73)	21.73 (11.76)	0.041
*T* (°C)	37.36 (0.84)	38.03 (0.37)	0.019
CO (L/min)	3.68 (0.69)	3.46 (0.54)	0.376
PAP (mmHg)	18.75 (3.7)	21 (3.2)	0.126
MAP (mmHg)	104.5 (18.1)	95.2 (19.5)	0.237
LAP (mmHg)	9.3 (2.5)	8.8 (2.5)	0.63
HR (beats/min)	68.33 (9.93)	81.67 (23.05)	0.079

Abbreviations: CO, cardiac output; HR, heart rate; LAP, mean left atrial pressure; PAP, mean pulmonary artery pressure; PVR, pulmonary vascular resistance; *T*, temperature; VV‐ECMO, veno‐venous extracorporeal membrane oxygenation.

^a^ Mean (standard deviation [SD]).

### 3.1 Part 1: Hyperoxia in the pulmonary vein did not change PVR

Exposing blood in the pulmonary vein to hyperoxia did not have a statistically significant impact on PVR (*p* = 0.686) or other values, except BP, which fell from 110 to 103 mmHg (*p* = 0.042). PaO_2_ went from 30.10 to 64.50 kPa when FiO_2_ changed from 0.6 to 1.00 (*p* < 0.001) (Table 3).

**TABLE 3:** Hyperoxia in the pulmonary vein.

	FiO_2_ 0.6: mean (SD)/median (IQR)	FiO_2_ 1.0: mean (SD)/median (IQR)	*p*‐value
PVR abs (dyn·s/cm^5^)	176.04 (141.22–265.56)	153.85 (117.06–315.83)	0.686
PVR (%)	1 (1–1)	0.87 (0.83–1.22)	0.686
PaO_2_ (kPa)	30.10, (27.15–40.95)	64.50 (38.25–68.30)	0.8
PvO_2_ (kPa)	6.92 (0.84)	6.51 (1.03)	0.622
PaCO_2_ (kPa)	5.13 (0.65)	5.32 (0.59)	0.287
CO (L/min)	4.08 (0.71)	3.74 (0.57)	0.064
PAP (mmHg)	20 (3.3)	19 (2.78)	0.338
BP (mmHg)	110 (87.00–131.5)	103 (83.5–108.5)	0.042
HR (beats/min)	71 (8.79)	69 (7.66)	0.611

Abbreviations: BP, blood pressure; CO, cardiac output; HR, heart rate; IQR, interquartile range; PAP, mean pulmonary artery pressure; PVR, pulmonary vascular resistance; SD, standard deviation.

#### 3.1.1 Hypoxia in the pulmonary vein doubled PVR

During hypoxia in the pulmonary vein, there were statistically significant changes in PVR, PaO_2_, PaCO_2_, and HR. The PVR absolute and PVR percentages increased to twice the baseline values, from 278.36 to 525.22 dyn·s/cm^5^ (*p* = 0.014) and from 100% to 207% (*p* = 0.022), respectively. PaO_2_ fell from 28.82 to 6.7 kPa (*p* = 0.007), and despite unchanged minute volume on the ventilator, PaCO_2_ increased from 4.83 to 6.10 kPa (*p* = 0.006). HR increased from 69 to 100 beats/min (*p* = 0.028). Median BP fell from 89.3 to 68.2 mmHg (*p* = 0.008). PAP increased from 20.5 to 33.8 mmHg (*p* < 0.001). pH_a_ decreased from 7.411 to 7.288 (*p* = 0.004). PvO_2_, CO, and lactate did not show significant changes (Table 4).

**TABLE 4:** Hypoxia in the pulmonary vein.

	FiO_2_ 0.6: mean (SD)/median (IQR)	FiO_2_ 0.05: mean (SD)/median (IQR)	*p*‐value
PVR (dyn·s/cm^5^)	278.36 (112.47)	525.22 (195.81)	0.014
PVR (%)	100 (0)	207 (81)	0.022
PaO_2_ (kPa)	28.82 (13.09)	6.7 (1.34)	0.007
PvO_2_ (kPa)	10.64 (6.20)	8.40 (2.98)	0.474
PaCO_2_ (kPa)	4.83 (0.71)	6.10 (0.59)	0.006
CO (L/min)	3.29 (0.59)	4.01 (1.06)	0.059
PAP (mmHg)	20.5 (3.4)	33.8 (4.7)	<0.001
BP (mmHg)	89.3 (20.0)	68.2 (14.4)	0.008
HR (beats/min)	69 (62–93)	100 (83–153)	0.028
pH_a_	7.411 (0.068)	7.288 (0.035)	0.004
Lactate (mmol/L)	1.06 (0.53)	2.34 (2.26)	0.229

Abbreviations: BP, blood pressure; CO, cardiac output; HR, heart rate; IQR, interquartile range; PAP, mean pulmonary artery pressure; PVR, pulmonary vascular resistance; SD, standard deviation.

**FIGURE 3:**
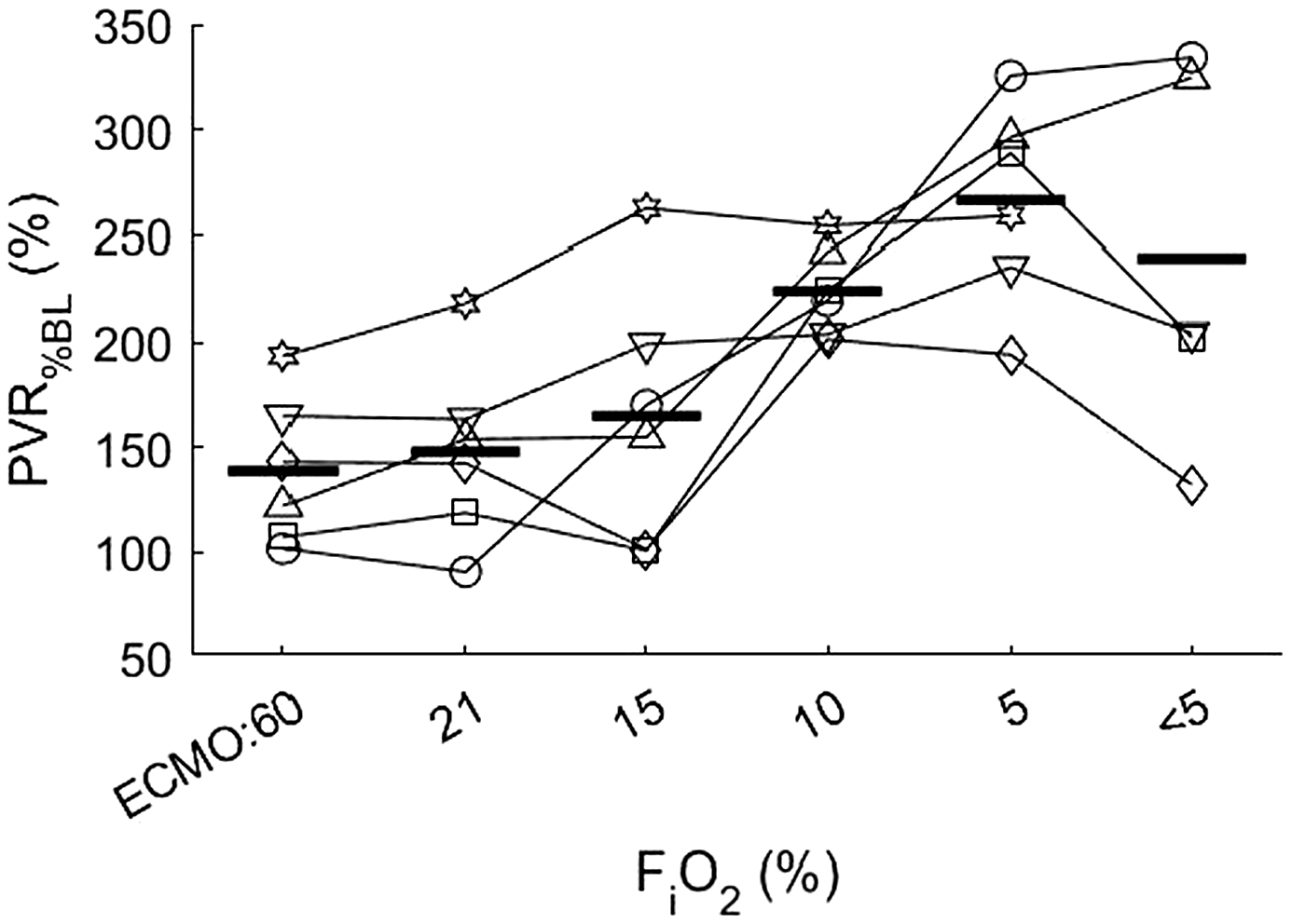
Change in pulmonary vascular resistance (PVR) relative to value at the start of extracorporeal membrane oxygenation (ECMO) in the individual pigs during the deoxygenation of pulmonary venous blood. Horizontal lines show mean response at each experimental step. Deoxygenation of the blood in the pulmonary vein caused a large increase in PVR in all pigs, up to threefold from the baseline value after establishment of veno‐venous (VV)‐ECMO.

Figure 3 shows how PVR, as a percentage of baseline values, increased during a stepwise decrease in FiO_2_ in the ventilator while maintaining high oxygenation in the pulmonary artery with VV‐ECMO. PVR peaked at an FiO_2_ of 0.05, after which it fell again in four pigs but continued to rise in two pigs.

### 3.2 Part 2: Hypoxia in the pulmonary artery produced no systematic change in PVR

**FIGURE 4:**
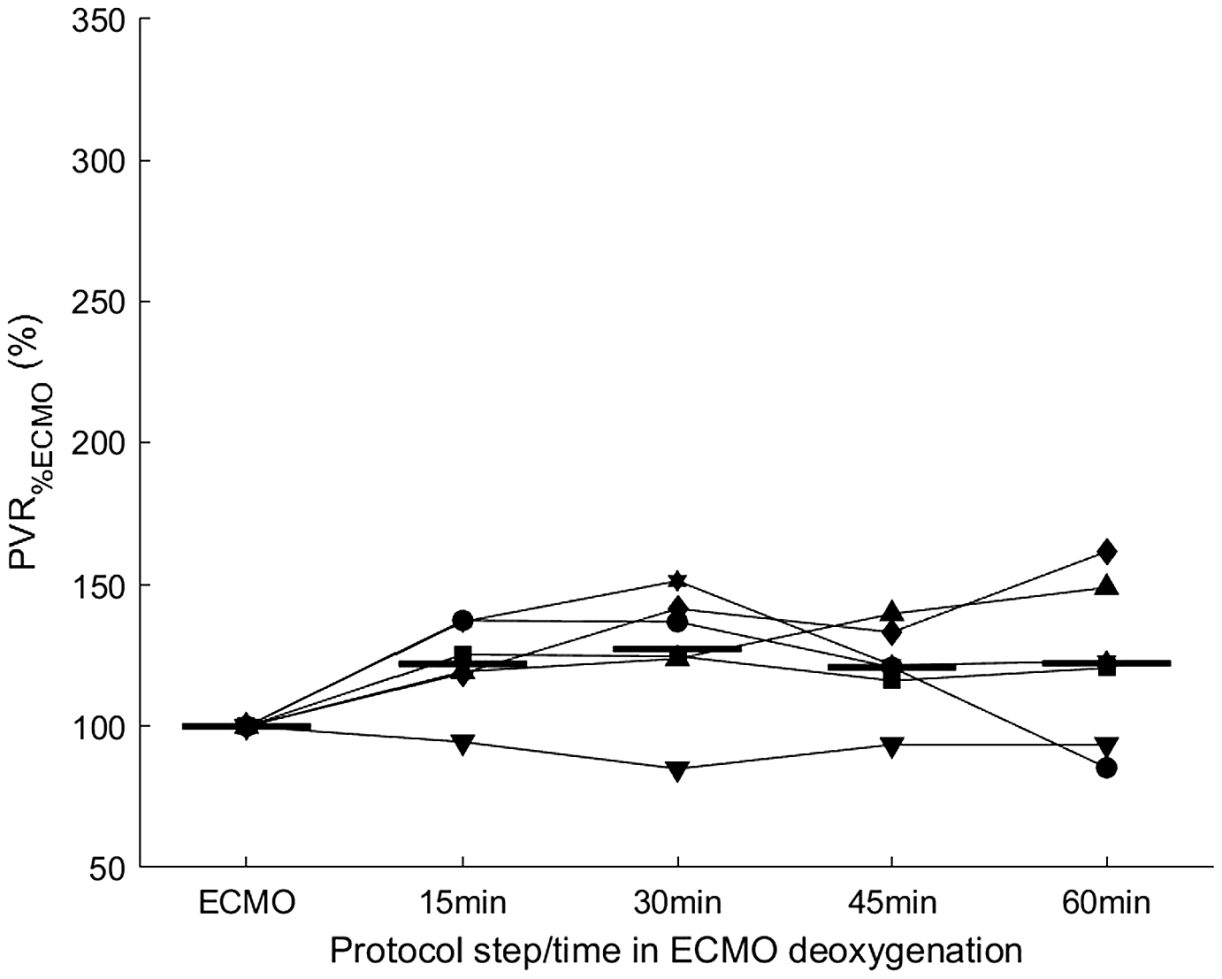
Change in pulmonary vascular resistance (PVR) relative to value at the start of extracorporeal membrane oxygenation (ECMO) in the individual pigs during hypoxia in the pulmonary artery over 1 h. Horizontal lines show mean response at each experimental step and for each consecutive 15 min of pulmonary arterial hypoxia.

PVR increased when the blood in the pulmonary artery was deoxygenated, but the increase was nonsignificant (*p* = 0.191). PaO_2_ fell from 13.6 to 11.85 kPa (*p* = 0.026), and PvO_2_ decreased from 5.47 to 3.67 kPa (*p* < 0.001). Although both changes were significant, PaO_2_ remained within the normal physiological range. Other measured parameters showed insignificant changes (*p* > 0.05; Table 5). During hypoxia in the pulmonary artery, PVR did not vary by a substantial amount, and none of the changes were significant. All pigs showed changes of less than 75% from the ECMO baseline after 1 h with low oxygen while on ECMO (Figure 4). The results in table four and five covers a prolonged period described in detail in the supporting informations.

**TABLE 5:** Hypoxia in the pulmonary artery.

	ECMO baseline: mean (SD)/median (IQR)	ECMO 1 hour: mean (SD)/median (IQR)	*p*‐Value
PVR absolute (dyn·s/cm^5^)	302.71 (95.13)	348.79 (42.96)	0.191
PVR (%)	100 (0)	122 (30)	0.128
PaO_2_ (kPa)	13.60 (12.3–16.3)	11.85 (11.3–12.1)	0.026
PvO_2_ (kPa)	5.47 (0.42)	3.67 (0.36)	<0.001
PaCO_2_ (kPa)	5.31 (4.53–5.44)	5.04 (4.58–5.53)	0.824
CO (L/min)	3.63 (0.47)	3.29 (0.37)	0.143
PAP (mmHg)	21.5 (3.2)	23.8 (3.0)	0.201
BP (mmHg)	101.0 (18.8)	83.7 (17.7)	0.029
HR (beats/min)	79 (70–98)	76 (66–101)	0.752
pH_a_	7.403 (0.063)	7.371 (0.067)	0.103
Lactate (mmol/L)	0.78 (0.26)	1.05 (0.49)	0.053

Abbreviations: BP, blood pressure; CO, cardiac output; ECMO, extracorporeal membrane oxygenation; HR, heart rate; IQR, interquartile range; PAP, mean pulmonary artery pressure; PVR, pulmonary vascular resistance; SD, standard deviation.

## 4 DISCUSSION

In this study, we demonstrated a novel method to measure and evaluate changes in PVR in anesthetized pigs. Using the ECMO technique and advanced ventilator adjustments allowed us to create extreme hypoxic situations in the pulmonary artery while maintaining normal oxygen pressure in the pulmonary veins and vice versa. Other factors known to influence PVR, such as pH, PaCO_2_, temperature, and CO, were kept within the normal range as much as possible.^20^

The setup was technically difficult with an open thorax and with a flow probe around the main pulmonary artery. In the daily clinic, many of the measurements could have been done less invasively with a pulmonary artery catheter, often called a Swan–Ganz (SG) catheter. In another study comparing SG and a flow probe in a porcine experiment, it took around 8 min for the SG measurements to detect at least a 30% decrease in CO with sudden massive bleeding.^21^ In experiments with severe hypoxia or toxicology testing, the animal could theoretically die before SG detects the problem.

In contrast to the SG method, the flow probe measures continuously, and in our setup there were continuous measurements of BPs on both sides of the pulmonary circulation, and blood analysis could be made from both sides via the catheters in the pulmonary artery and the left atrium.

To some extent the novelty of the experiment can be discussed, because in 1974 Smith et al. already performed an experiment in dogs with a VV‐ECMO system using both oxygenation and deoxygenation, but, of course, the oxygenators were not as effective as they are now.^22^ A similar experiment was also performed by Bishop in dogs in 1983.^23^ In 1983 Domino performed experiments in dogs with an open thorax and the use of a VV‐ECMO system and also with flow probes.^24^ The purpose of these experiments was to examine the influence of oxygen in the pulmonary artery and of CO on intrapulmonary shunting during different interventions on parts of the lungs.

There was no statistically significant difference between the 12 pigs used for the experiments. Their baseline PVR values were somewhat dispersed, but that was overcome by using the percentage increase or decrease in PVR.

PVR always increased when the animals were coupled to VV‐ECMO. There are multiple reasons that could explain this. The inflammatory response caused by ECMO is a reaction to the exposure of the blood to extracorporeal circulation. In our case, the ECMO tubes were not primed with blood but with saline, and the inflammatory response was augmented because of hemodilution, which led to increased neutrophil activation. The vascular endothelium plays a large role in the inflammatory response during ECMO. Activated endothelial cells secrete proinflammatory cytokines.^25^

The animals' body temperature increased when coupled to ECMO, but the temperature was kept constant with the use of a heating–cooling device. The temperature rose by slightly less than 1°C on average.

Fluid replacement was performed according to existing guidelines for porcine anesthesia to ensure normal rehydration and maintenance of fluid balance.^26^ However, these recommendations are only for anesthetized animals and not for animals on ECMO, where capillary leak is a known problem.^27^ Therefore, when too little flow in the tubes resulted in rattling, extra saline was given to address this. There was no use of vasoactive medications.

### 4.1 Hyperoxia in the pulmonary vein

The only parameter that changed significantly during hyperoxia in the pulmonary vein was the mean arterial pressure, which fell from 110 to 103. This is the opposite of what would be expected, as hyperoxia in the systemic circulation would cause vasoconstriction and thereby increase BP. PVR fell by approximately 13%, showing a very modest, nonsignificant decrease with increasing oxygen fraction. The other parameters measured were stable. These results are consistent with earlier reports of hyperoxic effects on PVR in both pigs and humans.^28^, ^29^

### 4.2 Hypoxia in the pulmonary vein

When FiO_2_ was decreased in the ventilator, PVR increased steadily during FiO_2_ values of 0.15, 0.10, and 0.05, but it was more pronounced for three of the animals. PAP increased in the same manner, steadily during the changes in FiO_2_. When FiO_2_ was under 0.05, PVR fell in four animals and continued to increase in two animals. There was a slight nonsignificant increase in CO, which may also have contributed to the increase in PVR. The PaO_2_ was between 5.41 and 6.88 kPa during the last measurements. This decrease in PVR during the most extreme hypoxemia might indicate some kind of hypoxic pulmonary vasodilation, as observed in experiments with isolated lungs where vasodilatation occurred if PaO_2_ was below 6.67 kPa.^17^ We cannot identify if this was due to the failure of vascular tone caused by extreme hypoxia. PvO_2_ was held normoxic with VV‐ECMO. There was a decrease from 10.64 to 8.40 kPa when the venous blood from the pulmonary artery entered the lungs, indicating deoxygenation in the alveoli due to the ventilation with extremely low FiO_2_. This deoxygenation could explain some of the results from other experiments in dogs and pigs performed by Domino in 1983 and by Mendes in 2022, where ECMO gave high SvO_2_ in the pulmonary artery but still with reduced blood flow to nonventilated parts of the lungs.^24^, ^30^

An increase in HR, together with a lowering in BP, is a normal response to hypoxia, which is a very strong stimulus to the sympathetic nervous system. The increase in PaCO_2_ indicated pulmonary shunting augmented by increasing CO, indicating the worsening of lung function, and ended very close to the upper level for normal PaCO_2_ values. This might interfere with PVR, as hypercapnia is a well‐known stimulus for HPV.^31^ It would have been better if we had increased the sweep flow to the ECMO system. Balanos et al. showed an approximate increase of 0.08–0.13 mmHg in the pulmonary vasculature for every 0.13 kPa increase in PaCO_2_, and because the values in our experiments were not far above the normal reference values for PaCO_2_, it is more likely that the increase in PVR was in fact due to hypoxia and not hypercapnia.^32^ pH decreased significantly and might have affected the increase in PVR because acidosis is known to affect HPV. Gordon et al. state that acute acidosis will not increase PVR significantly, but the effect will be significant with sustained acidosis,^33^ whereas Brimioulle et al. did not see any changes in HPV during respiratory acidosis in anesthetized dogs.^34^

### 4.3 Hypoxia in the pulmonary artery

The PVR increased by approximately 22% during the first hour of hypoxia in the pulmonary artery, although the increase was not statistically significant. PvO_2_ changed significantly during the first hour of hypoxia. PaO_2_ also fell 1.8 kPa during the first hour but was still within the normal range; therefore, this was not the reason for the increased PVR, because changes in PVR due to hypoxemia are not represented until the PaO_2_ is below the lowest normal value, 10.5 kPa. These findings correlate with the results of Holzgraefe et al., where alveolar normoxia regulated PVR independently of PvO_2_.^35^ This might indicate that the regulation of PVR does not primarily occur in the pulmonary artery, but we cannot rule out that some of the regulation might take place there, perhaps with retrograde communication, as suggested by Wang et al.^8^

### 4.4 Strengths and limitations

The experiments were conducted on young and healthy pigs with normal lung function. Each pig served as its own control, minimizing the possible individual variation that could add confounding to the data. A major limitation is, of course, the species difference when the results are extrapolated to humans. Another limitation is that all animals were young with healthy lungs without atelectasis. We used only female pigs for logistic reasons. A disadvantage of our present study is that it is not possible to extremely lower oxygenation in the pulmonary veins because it will give the same low PaO_2_ and kill the animal.

An open thorax might influence PVR because alveolar vessel resistance increases and extra‐alveolar vessel resistance decreases with increasing lung volume. However, it was essential for the PVR calculations to use an open thorax model and a flow probe around the pulmonary artery to obtain reliable results, as VV‐ECMO could also affect the measurements with an SG catheter due to thermodilution.^21^, ^36^, ^37^, ^38^

## 5 CONCLUSION

In this animal model with hypoxic conditions in the pulmonary artery, while maintaining normal oxygenation in the pulmonary vein and vice versa, very low PvO_2_ in the pulmonary artery did not induce a systematic increase in PVR to such an extent that PvO_2_ could be concluded to be the major regulator of PVR. During alveolar hypoxia with extremely low FiO_2_, ECMO and hyperoxia in the pulmonary artery could not attenuate systemic hypoxia and reduce PVR. The results support the theory that HPV is triggered by a lack of oxygen on the venous side of the alveoli.

### 5.1 Perspectives

It is of clinical interest to know exactly where PVR is regulated. It is possible to enhance pulmonary artery oxygenation with VV‐ECMO, which will enhance pulmonary venous oxygenation if no deoxygenation occurs in the lungs during ventilation. This could theoretically happen at high altitudes or during the aeromedical evacuation of patients. Breathing pure oxygen for a prolonged period seems to be harmful to the lungs, and even though hypoxia induces HPV, it does not mean that high doses of oxygen are beneficial.^39^

## AUTHOR CONTRIBUTIONS

Benedict Kjaergaard conceived the main research idea and supervised the project. Sigridur Olga Magnusdottir, Carsten Simonsen, and Benedict Kjaergaard performed the surgeries together, collected all data, and performed blood gas analyses. Sigridur Olga Magnusdottir and Dan Stieper Karbing performed the statistical analysis. Sigridur Olga Magnusdottir processed and performed data analysis. Sigridur Olga Magnusdottir interpreted the results and wrote the first draft of the paper. All authors assisted in drafting the manuscript and approved the final document.

## FUNDING INFORMATION

This study was funded by Aalborg University Hospital.

## CONFLICT OF INTEREST STATEMENT

The authors have no conflicts of interest.

## ETHICS STATEMENT

The experiments were carried out according to Danish and European legislation. The experiments were approved by the Danish Animal Experiment Inspectorate, license no. 2016‐ 15‐ 0201‐ 00930.

## Supporting information


Data S1.

Table S4.

Table S5.
