# Supplementary material for: Hypoxia in the pulmonary vein increases pulmonary vascular resistance independently of oxygen in the pulmonary artery
Source: Animal Model Exp Med. 2024 Mar 20;7(2):156–65. doi: 10.1002/ame2.12402 (PMC11079156; doi:10.1002/ame2.12402)
Supplement: Supplementary file 1 — Data S1. [file AME2-7-156-s002.docx]

The results in table four and five covers a prolonged period described in detail in the supporting informations.
