# Supplementary material for: Hypoxia in the pulmonary vein increases pulmonary vascular resistance independently of oxygen in the pulmonary artery
Source: Animal Model Exp Med. 2024 Mar 20;7(2):156–65. doi: 10.1002/ame2.12402 (PMC11079156; doi:10.1002/ame2.12402)
Supplement: Supplementary file 2 — Table S4. [file AME2-7-156-s003.pdf]

Hypoxia in the pulmonary vein increases pulmonary vascular resistance independently of oxygen in the pulmonary artery

A porcine study with extracorporeal membrane oxygenation and deoxygenation.

Online supplement

Table 4, extended version:

	**T0: FiO_2** 0.6 *	**T1: FiO_2** 0.8*	**T2: FiO_2** 1.0*	**T3: FiO_2** 0.4*	**T4: ECMO** *	**T5: FiO_2** 021*	**T6: FiO_2** 0.15*	**T7: FiO_2** 0.10*	**T8: FiO_2** 0.05*	**T9: FiO_2** < 0.05*
PVR (dyn·sec/cm^5)	197.36 (61.32)	171.82 (88.53)	203.92 (103.86)	238.51 (93.65)	278.36 (112.47)	301.11 (139.71)	334.35 (164.67)	445.60 (159.56)	525.22 (195.81)	490.79 (298.00)
PVR (%)					100 (0)	106.22 (12.93)	119.22 (33.48)	169.84 (42.21)	207.33(80.52)	199.76 (97.87)
PaO_2	30.02 (10.21)	42.83 (4.80)	63.38 (6.76)	23.70 [12.20-24.65]	28.82 (13.09)	13.09 (4.56)	9.63 (2.11)	7.88 (1.73)	6.7 (1.34)	5.89 (0.63)
PvO_2 (kPa)	6.57 (1.13)	6.82 (0.59)	6.51 (1.03)	5.68 (0.76)	10.64 (6.20)	10.27 (4.59)	8.78 (2.89)	8.66 (2.78)	8.40 (2.98)	8.68 (0.85)
$PaCo_2$ (kPa)	5.24 (0.64)	4.91 (0.93)	5.32 (0.59)	5.28 (0.49)	4.83 (0.71)	5.12 (0.77)	4.69 (0.97)	5.43 (0.54)	6.10 (0.59)	5.68 (1.13)
CO (L/min)	4.02 (0.66)	3.91 (0.68)	3.74 (0.57)	4.16 (0.68)	3.29 (0.59)	3.67 (0.91)	3.87 (0.77)	3.62 (0.65)	4.01 (1.06)	4.24 (1.70)
PAP (mmHg)	20 (3.0)	19 (2.9)	19.2 (2.8)	22.8 (4.6)	20.5 (3.4)	23.3 (5.4)	24.3 (4.4)	29.3 (5.7)	33.8 (4.7)	32.2 (6.7)
MAP (mmHg)	107.3 (24.1)	111.3 (6.8)	103.0 [83.5-108.5]	109.5 [91.8-115.8]	89.3 (20.0)	86.3 (15.9)	81.7 (20.4)	65.5 [62.8-90.0]	68.2 (14.4)	59.4 (16.0)
HR (beats/min)	70 (9)	73 (9)	69 (8)	72 (16)	69 [62-93]	84 (25)	95 (28)	96 (32)	100 [83-153]	109 (35)
pHa	7.424(0.065)	7.441 (0.047)	7.425 (0.065)	7.424 (0.063)	7.411 (0.068)	7.395 (0.063)	7.411 (0.076)	7.356 (0.052)	7.288 (0.035)	7.288 (0.035)
Lactate (mmol/L)	0.88 (0.25)	0.80 [0.73-1.48]	0.80 [0.70-1.88]	0.70 [0.65-1.45]	1.06 (0.53)	0.9 (0.47)	0.95 (0.52)	1.34 (0.84)	2.34 (2.26)	2.17 (1.32)

* mean (SD)/median [IQR]
