# Supplementary material for: Hypoxia in the pulmonary vein increases pulmonary vascular resistance independently of oxygen in the pulmonary artery
Source: Animal Model Exp Med. 2024 Mar 20;7(2):156–65. doi: 10.1002/ame2.12402 (PMC11079156; doi:10.1002/ame2.12402)
Supplement: Supplementary file 3 — Table S5. [file AME2-7-156-s001.pdf]

Table 5, extended version:

	**ECMO baseline (T1)***	**ECMO baseline (T2)***	**ECMO baseline (T3)***	**ECMO baseline (T4)***	**ECMO baseline (T5)***
PVR (dyn·sec/cm^5)	302.71 (95.13)	366.15 (111.34)	375.96 (105.18)	355.69 (77.14)	348.79 (42.96)
PVR (%)	100 (0)	122.08 (15.83)	127.33 (23.23)	120.92 (16.03)	122 (30)
PaO2 (kPa)	13.60 [12.3-16.3]	11.77 (1.53)	11.33 (1.73)	11.15 (0.92)	11.85 [11.3-12.1]
PvO2 (kPa)	5.47 (0.42)	4.29 (0.65)	3.85 (0.42)	4.18 (0.50)	3.67 (0.36)
PaCO2 (kPa)	5.31 [4.53-5.44]	4.8 (0.62)	4.88 (0.48)	4.98 (0.43)	5.04 [4.58-5.53]
CO (L/min)	3.63 (0.47)	3.58 (0.48)	3.43 (0.59)	3.38 (0.35)	3.29 (0.37)
PAP (mmHg)	21.5 (3.2)	24.8 (2.8)	26.2 (3.2)	24.2 (1.7)	23.8 (3.0)
MAP (mmHg)	101.0 (18.8)	97.7 (22.7)	93.5 (16.3)	92.33 (17.7)	83.7 (17.7)
HR (beats/min)	79 [70-98]	76.0 [67.75-109.50]	76.0 [72.75-102.75]	75.5 [66.00-101.00]	76.0 [66-101]
pHa	7.403 (0.063)	7.410 (0.079)	7.403 (0.073)	7.381 (0.067)	7.371 (0.067)
Lactate (mmol/L)	0.78 (0.26)	0.87 (0.31)	0.93 (0.35)	0.98 (0.50)	1.05 (0.49)

*Mean (SD) or median [IQR]
